# Stability of African Swine Fever Virus in Carcasses of Domestic Pigs and Wild Boar Experimentally Infected with the ASFV “Estonia 2014” Isolate

**DOI:** 10.3390/v12101118

**Published:** 2020-10-01

**Authors:** Melina Fischer, Jane Hühr, Sandra Blome, Franz J. Conraths, Carolina Probst

**Affiliations:** Friedrich-Loeffler-Institut, 17493 Greifswald-Insel Riems, Germany; Janehuehr@gmail.com (J.H.); Sandra.Blome@fli.de (S.B.); Franz.Conraths@fli.de (F.J.C.); Carolina.Probst@fli.de (C.P.)

**Keywords:** African swine fever, virus stability, tenacity, infectivity, risk factor, wild boar, carcass

## Abstract

Europe is currently experiencing a long-lasting African swine fever (ASF) epidemic, both in domestic pigs and wild boar. There is great concern that carcasses of infected wild boar may act as long-term virus reservoirs in the environment. We evaluated the tenacity of ASF virus (ASFV) in tissues and body fluids from experimentally infected domestic pigs and wild boar, which were stored on different matrices and at different temperatures. Samples were analysed at regular intervals for viral genome and infectious virus. ASFV was most stable in spleen or muscles stored at −20 °C and in blood stored at 4 °C. In bones stored at −20 °C, infectious virus was detected for up to three months, and at 4 °C for up to one month, while at room temperature (RT), no infectious virus could be recovered after one week. Skin stored at −20 °C, 4 °C and RT remained infectious for up to three, six and three months, respectively. In urine and faeces, no infectious virus was recovered after one week, irrespective of the matrix. In conclusion, tissues and organs from decomposing carcasses that persist in the environment for a long time can be a source of infection for several months, especially at low temperatures.

## 1. Introduction

Europe is currently experiencing a large and long-lasting African swine fever (ASF) epidemic, both in domestic pigs (*Sus scrofa domesticus*) and wild boar (*Sus scrofa*). ASF is an infectious disease of domestic pigs and wild boar that causes a hemorrhagic fever-like illness with an exceptionally high case fatality rate [1,2]. Between 2014 and the end of April 2020, over 25,100 ASF cases in wild boar were registered in the Animal Disease Notification System of the European Union [3].

Feeding contaminated pork meat and meat-derived products represents one of the major causes of disease transmission across countries [4]. Therefore, a series of studies have addressed the persistence of ASFV in pork products [5] including ham [6,7,8,9]. Given the high stability of ASFV in the environment, the concern about a further spread of ASF and its introduction into disease-free regions through contaminated pork products has risen [10,11]. 

Contaminated pork is also considered the main risk for ASFV introduction into wild boar populations [12,13]. Once the virus has been introduced, the main transmission pathways are direct contact between wild boar and indirect contact of wild boar with carcasses of infected conspecifics, so that it can circulate in the population for a long time [14,15,16]. Wild boar have been observed scavenging [17] and chewing on the bones of conspecifics [18]. Based on field data from Eastern Poland, it was estimated that more than half of the transmission events were due to contact with an infectious carcass [19]. Therefore, fast localisation and removal of carcasses is considered as one of the most important disease control measures [12,20]. However, it has been estimated that considerable numbers of infected carcasses are not found or are inaccessible for safe disposal [14]. This means that most carcasses decompose in situ, where they are available to susceptible conspecifics.

While ASF is considered to be a habitat-borne disease [21], the exact localisation of infectious virus in body tissues or in the decomposition island that forms in the soil around the carcass, and the environmental conditions under which infectivity is maintained are still regarded as major research gaps in ASF epidemiology [22,23].

In the context of ASFV tenacity, a large body of publications/literature is usually cited, several of which represent secondary sources [12,24,25,26]. Only a few original studies have actually investigated the infectivity of ASFV and most of them were published at the beginning of the 20th century (Table 1). They determined ASFV tenacity by means of in vivo assays in preserved [27,28] or clotted blood [29,30] and confirmed that ASFV maintains infectivity in blood at 4 °C for as long as 18 months [31] or even six years “in the cold” [32]. By means of virus isolation, a recent study has shown that both blood and muscle can remain infectious for more than three months [33].

The environmental stability of the virus and its genome has also been analysed in different organs. By means of in vivo assays, it was shown that spleen buried in soil can remain infectious for up to 280 days (i.e., about 10 months) [29]. Based on in vitro assays, organs are predicted to remain infectious for up to 714 days at −20 °C, 136 days at 4 °C and 17 days at 23 °C [34]. While the tenacity of ASFV in blood and inner organs is well studied, knowledge about the stability of infectious ASFV in bones and skin, i.e., organs and tissues that decompose slowly and persist in the environment for a long time, is limited. Such information is crucial to assess the risk of transmission of ASFV from carcasses within a wild boar population. A recent study analysed ASF-positive buried carcasses of wild boar, and detected stable quantities of ASFV genome, but no infectious virus [35].

Regarding excretions, in vivo experiments have shown that faeces and urine stored at 4 °C can remain infectious for up to 160 days and 60 days, respectively [29]. A more recent study estimated that the half-life of infectious ASFV ranges from 0.65 days in faeces stored at 4 °C to 0.29 days in faeces stored at 37 °C, and from 2.19 days in urine stored at 4 °C to 0.41 days in urine stored at 37 °C [36]. Regarding contaminated field crops, the probability of ASFV transmission has been estimated as low, if crops are stored in a dry place at room temperature for at least two hours [37]. ASFV is resistant to changes in pH, and certain strains have been reported to be resistant to complete inactivation at pH values between 4 and 13 [31]. In pig slurry, ASFV is relatively stable at 4, 22 and 40 °C. However, when heated to temperatures between 53 and 60 °C, the virus is inactivated to levels below the detection limit within 15 min [38]. Other authors have reported that ASFV is inactivated at 60 °C within 30 min [31]. Montgomery [27] noted that the sun in East Africa is a powerful disinfectant for ASFV. Apart from heat, the virus can also be inactivated by commercial disinfectants like citric acid or sodium hypochlorite [39] or lipid solvents [40].

The aim of this study was to determine the viability of ASFV during long-term storage on a variety of different matrices under several simulated environmental conditions to obtain information on its probable stability in natural habitats. The aim was to further elucidate the potential role of carcasses of ASFV-infected domestic pigs and wild boar in the epidemiology of ASF.

## 2. Materials and Methods

### 2.1. Study Design and Sample Collection

Infectious material was obtained from three domestic pigs (DP) and three wild boar (WB) that had been experimentally infected with the “Estonia 2014” strain [43]. In the animal experiment, all applicable animal welfare regulations including EU Directive 2010/63/EC and institutional guidelines were taken into consideration. The animal experiment was approved by the competent authority (Landesamt für Landwirtschaft, Lebensmittelsicherheit und Fischerei of the German federal state of Mecklenburg-Western Pomerania under reference number 7221.31-064/17). The animals were infected oro-nasally with 2 mL cell culture supernatant containing 10^5.25^ haemadsorbing units (HAU)/mL of ASFV. On day 7 post-infection, i.e., at the peak of viremia, the animals were euthanised. During necropsy, the skull, blood, spleen, bones (both limb bones and ribs), striated muscles (shoulder, back, tight), skin with skin fat, urine and faeces samples were collected.

**Figure 1:**
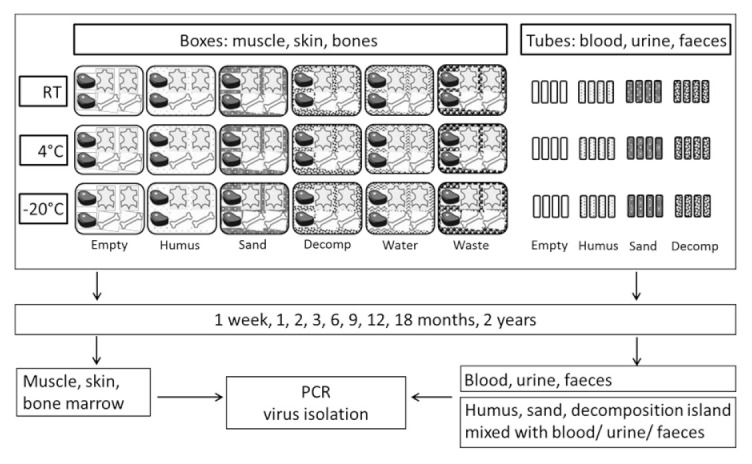
Study design. Storage of the samples of three domestic pigs (DP1–3) and three wild boar (WB1–3) alone (empty) or on different matrices: humus, sand, decomposition island (decomp), water or waste.

To mimic natural conditions, samples were stored on various matrices (humus, sand, decomposition island, water or mixed waste) at three different temperatures (−20 °C, 4 °C or room temperature) (Figure 1). Therefore, aliquots of 10 g humus, sand or soil from the decomposition island were placed into 50 mL Falcon tubes and spiked with 1 mL blood, urine or faeces of ASFV-infected pigs or wild boar, respectively. Bones, pieces of muscle or skin (approximately 15 × 15 cm) were placed on the respective matrices in plastic boxes (boxes for bones: 40 × 30 × 19 cm; boxes for muscle and skin: 59 ×39 × 43 cm; IKEA, Rostock, Germany). The boxes were either empty or filled with tap water or a thick layer of humus, sand, soil from the decomposition island or mixed waste, respectively, as matrices. We used commercially available humus (70% bog peat, pH 5.8, toom Baumarkt, Neuenkirchen, Germany) and sand (0–1 mm grain size, pH 6.6, toom Baumarkt, Neuenkirchen, Germany). Material of a decomposition island was recovered from underneath a decomposing carcass of an adult wild boar. The decomposition island is a highly concentrated area of fertility underneath and surrounding a carcass that is formed by cadaveric materials and metabolic products of the organisms that decompose the carcass [44]. It had a pH of 3.2 when collected. Waste was simulated by mixing different types of cheese (both whole portions and grated), sausages, yoghurt, cream, marmalade, bread, eggs, apples, peas and tomatoes including the empty packing materials freshly obtained from a local supermarket (Marktkauf, Neuenkirchen, Germany).

All tubes and boxes were labelled with consecutive numbers and the unique identifier of the respective animal (DP1–3, WB1–3). They were stored at −20 °C (DP1, WB1), 4 °C (DP2, WB2) or room temperature (RT) (DP3, WB3). The day of necropsy was considered the starting day of sampling (day 0), from which all sampling times were calculated. Samples were analysed on day 0, after one week and after 1, 2, 3, 6, 9, 12, 18 and 24 months for the presence of viral genome and infectious virus (Figure 1). Taking into account the limited stability of carcass material under natural conditions at ambient temperature (21 °C), RT samples were stored for only 3–6 months. At the indicated time points, pea-sized (approximately 6–8 mm in diameter) pieces of muscle, skin fat, spleen and bone marrow were taken from the whole pieces of tissue or from opened bones. Muscle samples were taken from the centre of thick areas to obtain the moistest sample. Skin fat samples were taken directly from beneath the skin. The whole pieces of tissues and cracked bones were disposed of immediately after sampling, i.e., each time a new piece of muscle and skin or a new bone was sampled.

In addition, matrix from underneath the pieces of muscle and skin (humus, sand and decomposition island) as well as the water, where the bones were stored in, were also sampled after 2, 3, 6 and 9 months.

Muscle, skin fat, spleen and bone marrow samples were homogenised in 1 mL sterile phosphate buffered saline (PBS) using a 5 mm steal bead in a TissueLyser II (Qiagen, Hilden, Germany) for three minutes at 30 Hz and afterwards centrifuged at 10,600× *g* for two minutes. The supernatants were used for further analysis. Blood, urine, faeces and matrix samples were processed according to a soil-optimised protocol [45]. Briefly, 10 mL of cell culture medium (RPMI-1640; Thermo Fisher Scientific, Schwerte, Germany) was added to each sample. Samples were vortexed thoroughly, sonicated (5 pulses, duty cycle 80%, output 8) and centrifuged at 1250× *g* for two minutes. The supernatant was then poured over a coffee or tea filter and subsequently passed through a syringe filter (0.45 µm Millex Filter Units; Merck Millipore Ltd., Tullagreen, Ireland). Two aliquots of each sample were stored at −80 °C until further processing.

### 2.2. Laboratory Analyses

The presence of ASFV DNA was detected by real-time quantitative polymerase chain reaction (qPCR). Prior to qPCR, viral nucleic acid was extracted using the NucleoMag VET kit (Macherey-Nagel, Düren, Germany) on the KingFisher 96 flex platform (Thermo Fisher Scientific, Schwerte, Germany) according to the manufacturer’s recommendations. Subsequently, nucleic acid was analysed by qPCR according to the protocol published by [46] in combination with an internal control based on an EGFP detection system [47] on a Biorad CFX real-time cycler (Bio-Rad Laboratories, Hercules, USA). Results of the qPCR were recorded as quantification cycle (Cq) values as determined by the Biorad CFX software. Using a dilution series of an ASFV DNA standard, the number of ASFV genome copies in the respective samples was determined.

To detect infectious virus, samples were tested using the haemadsorption test (HAT) as published previously [37]. When cytotoxicity of samples was observed, these were prediluted 10-fold in medium and tested again. Furthermore, end-point titrations were performed for selected samples including bone marrow and skin, as well as specimens that were positive over the entire study period (24 months). Therefore, two independent titrations were performed per sample at appropriate time points and mean values as well as standard deviation (SD) were calculated in Excel version 2019 (Microsoft GmbH, Unterschleißheim, Germany). For this setup, the limit of detection was set below 1.75 log_10_ 50% haemadsorbing doses per ml (HAD_50_/_mL_) due to the initial dilution and the number of inoculated wells. This corresponds to six haemadsorbing units in the 100 µL sample volume. To increase sensitivity for weak positive samples, HAT results of undiluted specimens were also considered for calculating virus titres. Titres were calculated using the Reed and Muench method [48] to determine the haemadsorbing dose (HAD); titres were expressed as log_10_ HAD_50_/_mL_.

## 3. Results

**Figure 2:**
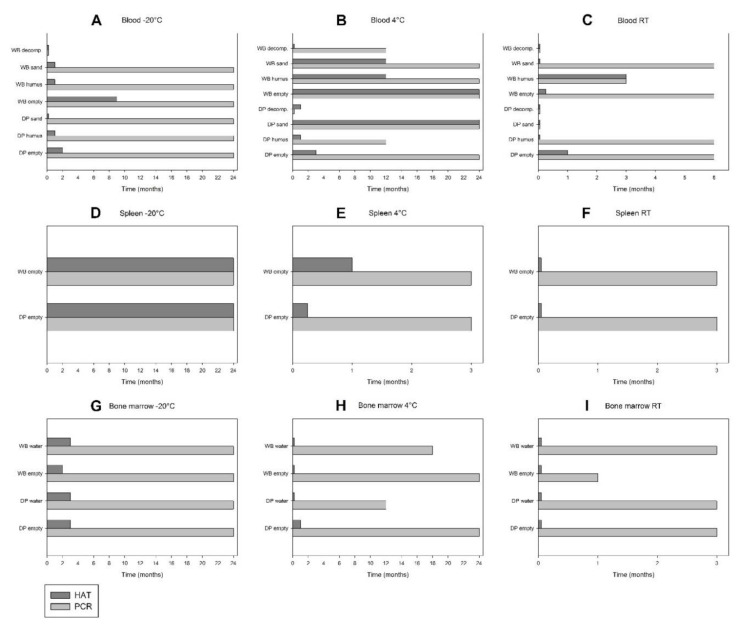
Comparison between qPCR and HAT results for blood (**A**–**C**), spleen (**D**–**F**) and bone marrow (**G**–**I**) of domestic pigs (DP) and wild boar (WB) during storage in empty boxes or on different matrices (humus, sand, decomposition island [decomp.], waste, water) at −20 °C, 4 °C and room temperature (RT).

**Figure 3:**
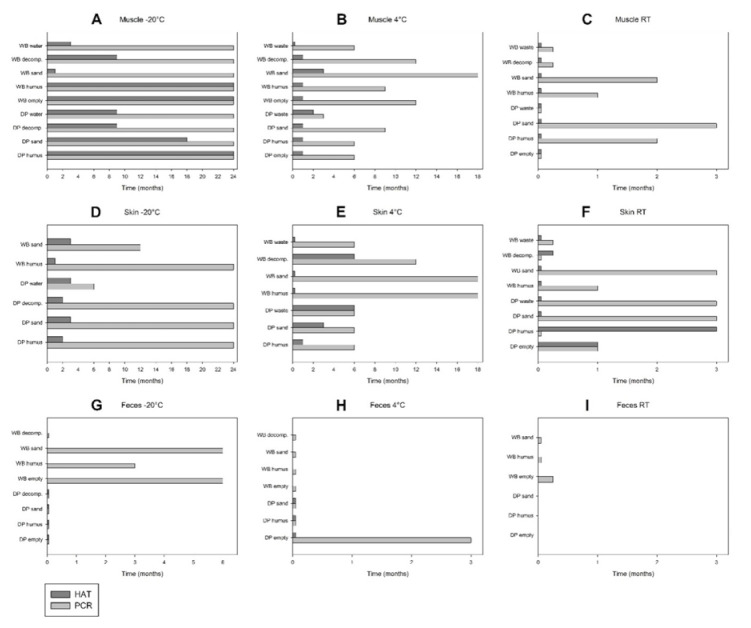
Comparison between qPCR and HAT results for muscle (**A**–**C**), skin (**D**–**F**) and faeces (**G**–**I**) of domestic pigs (DP) and wild boar (WB) during storage in empty boxes or on different matrices (humus, sand, decomposition island [decomp.], waste, water) at −20 °C, 4 °C and room temperature (RT).

To investigate the tenacity of ASFV, different body tissues, organs and fluids were stored at −20 °C, 4 °C or RT either alone or in different matrices (humus, sand, decomposition island, waste, water). At day 0, in all blood, spleen, bone marrow, muscle and skin samples, both ASFV genome and infectious virus were detected by PCR and HAT (Figure 2 and Figure 3). Blood and spleen displayed the highest number of genome copies, indicating the highest viral loads (Table 2). Regarding excretions, ASFV genome was detected in faeces and urine of all animals except DP3. Genome loads in faeces from WB were higher than in those from DP. Infectious virus was recovered from two faecal samples (DP1 and DP2) and in the urine of all animals except DP3 and WB3 (Table 2).

In blood stored at −20 °C, ASFV genome was detected for up to 24 months in six samples (DP and WB empty, humus, sand) (Figure S1A and Figure 2A). Infectious virus was detected for up to nine or two months in WB empty or DP empty, respectively, and for up to one month in DP humus, WB humus and WB sand. Remarkably, neither ASFV DNA nor infectious virus was recovered in WB decomposition, neither after one month nor later on.

**Figure 4:**
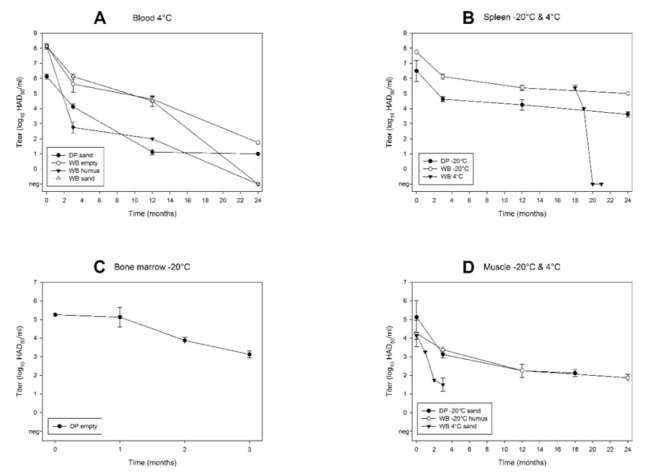
Kinetics of ASFV titres. (**A**) Blood stored at 4 °C (DP sand, WB empty, humus and sand); (**B**) spleen stored at −20 °C for 24 months (DP1, WB1) or at −20 °C until month 18, and at 4 °C during months 18–21 (WB2); (**C**) bone marrow from a bone stored at −20 °C in an empty box (DP empty); (**D**) muscle stored at −20 °C (DP sand, WB humus) and at 4 °C (WB sand). Individual data points represent mean values (± SD) from two independent titrations.

At 4 °C storage, ASFV genome was detected for up to 24 months in five samples (DP and WB empty, WB humus, DP and WB sand) and for up to 12 months in DP humus and WB decomposition (Figure S1B and Figure 2B). In DP decomposition, infectious virus but no ASFV genome could be detected. It is noteworthy that several DP samples displayed a large variation in the number of genome copies. **Infectious virus** was detected for up to 24 months in DP sand and WB empty and for up to 12 months in WB sand and WB humus (Figure 2B). Of these long-term positive blood samples, WB humus and WB sand displayed the largest decrease in viral titres (from initially 8.13 log_10_ to negative after 24 months) (Figure 4A). In WB empty, the titre dropped from about 8.13 log_10_ at day 0 to 1.75 log_10_ HAD_50_/_mL_ after 24 months. In DP sand, the titre dropped from initially 6.13 log_10_ to 1.00 log_10_ HAD_50_/_mL_ after 24 months. In blood stored at **RT**, the viral load (genome copy numbers) clearly decreased over time (Figure S1C). ASFV genome could be detected during the entire observation period (i.e., six months) in three samples (DP and WB empty, WB sand) and for up to three months in WB humus. **Infectious virus** was detected for up to three months, one month or one week in WB humus, DP empty or WB empty, respectively (Figure 2C).

In **spleen** stored at **−20 °C**, both ASFV genome and infectious virus were detected during the entire study period (i.e., 24 months) (Figure S1D and Figure 2D). While the viral load remained relatively constant (Figure S1D), the virus titre clearly decreased over time (Figure 4B). In WB spleen, a substantial titre loss from initially 7.75 log_10_ at day 0 to 5.00 log_10_ HAD_50_/_mL_ after 24 months was observed. During the first 18 months, all spleens were stored at −20 °C. After 18 months, pieces of spleen of DP2 and WB2 were stored at **4 °C** and **RT** for the following three months. In all these samples, ASFV genome was detected throughout the sampling period (Figure S1E,F). At 4 °C storage, **infectious virus** was detected for up to one month in WB2 spleen and for up to one week in DP2 spleen (Figure 2E). The titre loss in WB2 spleen was remarkable: After 18 months of storage at −20 °C, the ASFV titre was 5.38 log_10_ HAD_50_/_mL_. At month 19 (after one month storage at 4 °C), the titre of WB2 spleen had decreased to 4.00 log_10_ HAD_50_/_mL_, and at month 20 (after two months storage at 4 °C), viable virus was not detectable anymore (Figure 4B). At RT storage, the HAT was negative after one week (Figure 2F).

In **bones** stored at **−20 °C**, ASFV genome was detected in both red and yellow bone marrow of all samples during the entire observation period (i.e., 24 months) (Figure S1G). **Infectious virus** was detected for up to three months in three samples (DP empty, DP and WB water), and for up to two months in WB empty (Figure 2G). Bone marrow (red marrow) of DP had a virus titre of 5.25 log_10_ HAD_50_/_mL_ at day 0. Thus, the initial titre was about one log_10_ level lower than in the spleen of the same animal (6.50 log_10_) and about three log_10_ levels lower than in blood of WB at 4 °C (8.13 log_10_). Furthermore, within three months, bone marrow displayed 99% titre loss (from 5.25 log_10_ to 3.13 log_10_ HAD_50_/_mL_) (Figure 4C). At **4 °C** storage, ASFV genome was detected in bone marrow for up to 24 months in two samples (DP and WB empty), and up to 18 and 12 months in WB water and DP water each (Figure S1H and Figure 2H). **Infectious virus** was detected for up to one month in DP empty, while three samples were HAT-positive only on day 0 (DP water, WB empty, WB water) (Figure 2H). At **RT** storage, ASFV genome was detected until the end of the observation period (i.e., three months) in three samples (DP empty, DP water, WB water) and after one month in WB empty (Figure S1I and Figure 2I). No **infectious virus** was recovered after one week in any of the samples.

In **muscles** stored at **−20 °C**, ASFV genome was detected for up to 24 months in nine samples (DP and WB humus, DP and WB sand, DP and WB decomposition, DP and WB water, WB empty) (Figure S2A and Figure 3A). Remarkably, DP sand was PCR-negative in months 9 and 12, but positive in month 24. **Infectious virus** was recovered for up to 24 months in three samples (DP and WB humus, WB empty) and up to 18 months in DP sand (Figure 3A). In the muscle samples selected for titration (DP sand, WB humus), the titres dropped from 5.13 or 4.25 to 2.13 or 1.88 log_10_ HAD_50_/_mL_, respectively (Figure 4D). At **4 °C** storage, ASFV genome was detected during the entire observation period (i.e., 18 months) in WB sand and for up to 12 months in WB empty and WB decomposition (Figure S2B and Figure 3B). **Infectious virus** was recovered for up to three or two months in WB sand and DP waste each (Figure 3B). In the muscle samples selected for titration, the most rapid drop in virus titre was observed in WB sand at 4 °C, from initially 4.13 to 1.50 log_10_, over three months (Figure 4D). At **RT** storage, ASFV genome was detected until the end of the observation period (i.e., three months) in DP sand and for up to two months in DP humus and WB sand (Figure S2C and Figure 3C). Remarkably, DP sand displayed an increasing number of genome copies over time (Figure S2C). **Infectious virus** was not detected in any of the muscle samples at RT (Figure 3C) except on day 0.

In **skin** stored at **−20 °C**, ASFV genome was detected during the entire observation period (i.e., 24 months) in four samples (DP and WB humus, DP sand, DP decomposition) and for up to 12 months in WB sand (which was not tested any more after 12 months due to limited sample availability) (Figure S2D and Figure 3D). **Infectious virus** was recovered for up to three months in DP sand, DP water and WB sand (Figure 3D). At **4 °C** storage, ASFV genome was detected until the end of the observation period (i.e., 18 months) in two samples (WB humus, WB sand) and for up to 12 months in WB decomposition (Figure S2E and Figure 3E). **Infectious virus** was recovered for up to six months from DP waste and WB decomposition, and for up to three months from DP sand (Figure 3E). 

At **RT** storage, ASFV genome was detected until the end of the observation period (i.e., three months) in DP sand, DP waste and WB sand (Figure S2F and Figure 3F). **Infectious virus** was recovered for up to three months, one month or one week in DP humus, DP empty or WB decomposition, respectively (Figure 3F). Remarkably, DP humus and WB decomposition were longer HAT-positive than PCR-positive.

In **faeces** stored at **−20 °C**, ASFV genome was detected until the end of the observation period (i.e., six months) in two samples (WB empty, WB sand) and for up to three months in WB humus (Figure S2G and Figure 3G). From faeces stored at −20 °C, no **infectious virus** was recovered, irrespective of the matrix. At **4 °C** storage, ASFV genome was detected until the end of the observation period (i.e., three months) in DP empty (Figure S2H and Figure 3H). The remaining six samples were PCR-negative throughout the storage period. No **infectious virus** was recovered from faeces stored at 4 °C, irrespective of the matrix. At **RT** storage, ASFV genome was detected after one week in WB empty (Figure S2I and Figure 3I). **Infectious virus** could not be recovered.

In **urine** stored at **−20 °C**, ASFV genome was detected for up to six months in three samples (DP and WB sand, WB humus) and for up to three months in DP humus (Table S1). The two remaining samples (DP and WB decomposition) were PCR-negative throughout the storage period. From urine stored at −20 °C, no **infectious virus** was recovered, irrespective of the matrix. In urine stored at **4 °C**, ASFV genome was detected during the entire observation period (i.e., three months) in WB sand. The remaining two samples (WB humus, WB decomposition) were PCR-negative during storage at 4 °C. At **RT** storage, ASFV genome was detected after one week in WB empty.

From the **matrix** stored at **−20 °C**, **infectious virus** was recovered for up to two months in humus underneath DP muscle and the decomposition island underneath DP skin (Table S1). In the matrix stored at **4 °C**, ASFV genome was detected for up to nine months in three samples (sand underneath WB muscle, water containing DP or WB bones) and for up to six months in humus underneath DP muscle and sand underneath WB skin. At **RT** storage, ASFV genome was detected for up to six months in water containing DP or WB bones. A considerable amount of mould formed especially on the tissues stored on material of the decomposition island and on waste at 4 °C or RT (Figure S3).

## 4. Discussion

In this study, we assessed ASF virus and genome stability in various tissues, organs and fluids of carcasses of experimentally infected pigs and wild boar. Storage temperatures of −20 °C, 4 °C and RT and different matrices were chosen to mimic the four seasons in a temperate climate and typical deathbeds of wild boar in Central Europe. Since Germany was free of ASF at that time, we performed the study under laboratory conditions (biosafety level 3) and could not simulate the decomposition process of carcasses in a natural environment. The natural decomposition process of carcasses in the field is influenced by a large number of environmental variables such as sunlight, wind, rain, humidity or leaf litter, and biological factors like scavengers and necrophagous insects including larval secretions and metabolic products. In our study, we had to choose among the most important environmental variables (temperature and matrix) due to the fact that we had only a limited number of infected animals to obtain samples from. Furthermore, under laboratory conditions, it was not feasible to preserve entire carcasses, but only parts of them (e.g., bones or pieces of muscle or skin). Carcasses are complex structures consisting mainly of organic and some inorganic material, and body parts that have been separated from surrounding tissues provide micro-conditions that may differ from those in a body with intact tissue assemblies. Factors that may differ between a whole body in a natural environment and body parts under laboratory conditions are pH and temperature. Previous studies have observed significant biochemical alterations, including pH, during the decomposition process of pigs [49,50]. Larval masses of necrophagous insects produce thermal energy that makes them significantly warmer than ambient temperature [51]. In conclusion, the micro-conditions in a whole body decomposing in a natural environment differ from the micro-conditions in isolated body parts stored in an experimental setting. This may influence ASFV stability and should be taken into account when interpreting the results of this study.

Our results show that ASFV is most stable in spleen or muscles at −20 °C and in blood at 4 °C, from where infectious virus was recovered throughout the entire study period of two years. This finding suggests that a carcass of an infected pig or wild boar may remain infectious for at least two years under favourable conditions. Especially if adipocere, a chemical alteration of fatty tissue that may slow the decomposition rate or arrest the decomposition process altogether [52], is formed, tissues underneath containing ASFV may be protected from complete decay. Given the fact that internal organs like spleen are swiftly liquefied by putrefaction, metabolised by larvae or eaten by scavengers [53], the long-term survival under experimental conditions is probably not linked to long-term virus persistence under field conditions. Therefore, in contrast to Mazur-Panasiuk and Wozniakowski [34], who studied ASFV tenacity in spleen, lung and kidneys, we also studied tissues and organs that persist longer in the environment, especially at low temperatures, i.e., muscles, skin and bones.

Particularly at low temperatures (−20 °C), muscle samples remained infectious for the entire study period and for up to three months at 4 °C. Depending on the environment and the activity of necrophagous insects and scavengers, the skin can persist in the environment for even longer than muscles. In skin samples stored at 4 °C, viable virus was recovered for up to six months, and for up to three months at −20 °C or RT.

In marrow from bones stored in boxes in the absence of any matrix or in water, ASFV DNA could be detected for up to two years. However, an obvious decline in viable virus was observed after three months. In bones stored at −20 °C, viable virus was detected for up to three months, and at 4 °C for up to one month, while at RT, no infectious virus could be recovered after one week. There was a remarkable difference observed between bones rich in red marrow (i.e., ribs), and long bones rich in fatty yellow marrow such as the femur, humerus, radius and ulna. One possible explanation is that less ASFV is present in yellow bone marrow, mainly a reserve of fat cells. This observation shows that sampling ribs for ASFV detection might be more appropriate than long bones when a skeletonised carcass is found in the field. Potential differences between red and yellow bone marrow regarding the presence or detectability of ASFV so far have not been mentioned explicitly [54]. Our results also suggest that long bones rich in yellow marrow may be less important as reservoirs for ASFV regarding carcasses of infected pigs or wild boar. Bones rich in red marrow like ribs might be more important as a long-term source of infection. They are also thinner and thus can be readily cracked by large scavengers, so that they are likely to be consumed earlier.

Our study provides no evidence that the substrate of the carcass decomposition island is an important source of ASFV infection, since no viable virus could be recovered from blood mixed with soil of the decomposition island. The low pH measured in this matrix (initially pH 3.2) may have contributed to a rapid inactivation of ASFV. Moreover, viable virus could only be found in two matrix samples (humus underneath muscle and decomposition island under skin after two months at −20 °C). It has to be kept in mind, however, that filtering substrates with a high content of organic matter is difficult, which may lead to a decreased sensitivity of the detection assay (HAT). Under field conditions, it may be relevant how much blood the substrate contains, i.e., if the animal that died of ASF was wounded or injured and how long it bled on the deathbed, since ASFV seems to be preserved in blood for a long time. In relation to this, our results confirm that viable ASFV is highly stable in blood, even if it is mixed with sand or humus.

In urine and faeces, ASFV genome was detected for up to six months at −20 °C, up to three months at 4 °C and up to one week at RT. However, excretions were stored for a maximum of six, three and two months at the respective temperature because of their limited stability in the field, e.g., due to extensive mould growth on faeces. Remarkably, ASFV genome seems to be more stable in WB faeces than in faeces of domestic pigs at −20 °C (Figure 3G). The stability of ASFV DNA in faeces of domestic pigs has already been analysed by [41], who determined half-life values of more than two years at 4 °C and 22 days at RT (Table 1). In our study, no infectious virus could be recovered from urine and faeces after one week, irrespective of the storage temperature and the matrix. These findings are in accordance with the results of Davies et al. [36], who recovered infectious ASFV from urine for up to five days at 4 °C and RT and from faeces for up to five days if stored at 4 °C and for up to three days if stored at RT.

In general, the vast majority of the investigated samples were positive for ASFV genome for long periods. However, we observed a substantial fluctuation or variation in genome copy numbers over time, for example, within muscle samples obtained from the same animal, stored at 4 °C (Figure S2B). This finding indicates that the ASFV genome might not be homogeneously distributed in some body tissues. Nevertheless, ASFV DNA seems to be highly stable over time. Recently, Mazur-Panasiuk and Wozniakowski [34] obtained similar results, revealing that invariably all samples they investigated were qPCR-positive during a 112-days study with storage temperatures of −20, 4 and 23 °C.

In most cases, ASFV genome is detectable for much longer than infectious virus. Thus, a PCR-positive result must be interpreted with caution when it is used for assessing the ASFV infection risk. Interestingly, we found two samples (blood of DP stored with decomposition island at 4°C for one month, skin of DP stored on humus at RT for one to three months) which were negative for ASFV genome, but positive in the HAT. A possible explanation is that detection of infectious virus in swine macrophages is more sensitive than PCR. A recent study revealed that the HAT detection system was around two log_10_ levels more sensitive than the applied qPCR [55].

Although the HAT is a standard technique that is recommended to confirm ASFV-positive results obtained by other methods [56], its relative sensitivity is controversially discussed and has not been cross-validated sufficiently. Previous studies have shown that material that had previously been tested negative in the HAT could provoke clinical illness and death, if inoculated into pigs as a bioassay [6,7]. It might therefore be useful to confirm negative HAT results by bioassay, at least in samples of particular importance. In our study, inoculation of pigs was not an option due to the large number of animals that would have been necessary, also for statistical reasons.

## 5. Conclusions

This study confirms that tissues of pigs and wild boar that succumbed to ASF represent a long-term reservoir for ASFV. Especially at low temperatures, viable ASFV was detected for several months in all analysed tissues, irrespective of the matrix they were stored on. We therefore conclude that especially at lower temperatures, body parts like muscles, skin and bones that persist in the environment for a long time can remain a source of ASFV infection for several months. However, we observed a rapid decay of infectivity of ASFV in the urine and faeces of infected animals. Matrices like sand, humus, decomposition island or water, which were in contact with infectious material, seem to be less important as a reservoir for ASFV. The results highlight the need for safe carcass disposal to avoid the presence of infectious material in the environment, especially at low temperatures and if the animal that died of ASF was wounded or injured.

## Figures and Tables

**Table 1:** Primary literature regarding the tenacity of African swine fever virus (ASFV) in blood, different organs and body fluids.

Reference	Material	ASFV Stability	Method
**Blood**			
Montgomery 1921 [27]	Defibrinated blood (RT)	140 days	In vivo assay
	Preserved blood (RT)	404 days	
	Preserved blood (37 °C)	104 h	
	Preserved blood (40 °C)	15 days	
	Preserved blood (50 °C)	4 h	
	Preserved blood (55 °C)	1 h	
	Filtered serum (RT)	428 days	
Steyn 1932 [30]	Preserved blood in ice chests	12 months	In vivo assay
	Clotted blood from decomposed carcass	5 days	
Walker 1933 [28]	Preserved blood (1–4 °C)	1076 days	In vivo assay
De Kock et al., 1940 [32]	Blood stored “in a cold room in the dark”	6 years	In vivo assay
Kovalenko et al., 1965 [29]	Dried blood	2165 days	
	Native blood in sealed ampules	1526 days	
	Frozen meat (3–4 °C)	104 days	
Kovalenko et al., 1965 [29]	Fresh blood on wood, buried	81 days	In vivo assay
	Fresh blood on wood, surface	6 months	
	Fresh blood on bricks, buried	112 days	
	Clotted blood buried in garden soil	112 days	
	Clotted blood buried in sandy forest	81 days	
	Clotted blood in pond water	70 days	
Plowright and Parker 1967 [31]	Preserved blood (4 °C)	18 months	In vivo assay
Blome and Dietze 2011 [33]	Blood (4 °C, 18–22 °C, 37 °C)	3 months	Virus isolation
**Organs**			
Kovalenko et al., 1965 [29]	Spleen buried in soil	280 days	In vivo assay
Plowright and Parker 1967 [31]	Spleen suspension (−70 °C)	82–105 weeks	In vivo assay
	Spleen suspension (−20 °C)	marked instability	
Blome and Dietze 2011 [33]	Spleen (4 °C, 18–22 °C, 37 °C)	3 months	Virus isolation
de Carvalho Ferreira et al., 2014 [41]	Spleen, liver, lymph nodes, tonsil	Half-life: 4–80/2.7–7.4/1.8–4.6/1.7–3.8 days	PCR
Mazur-Panasiuk and Woźniakowski 2020 [34]	Spleen (−20 °C, 4 °C, 23 °C)	Predicted survival time: 483/126/15 days	PCR
	Putrescent spleen in water/soil/leaf litter/straw/hay/grain (4 °C)	Predicted survival time: 36/33/19/97/113/69 days	
	Lung (−20 °C, 4 °C, 23 °C)	Predicted survival time: 714/136/9 days	
	Kidney (−20 °C, 4 °C, 23 °C)	Predicted survival time: 353/35/17 days	
**Muscle**			
Blome and Dietze 2011 [33]	Muscle (37 °C)	3 months	PCR, virus isolation
	Muscle (4 °C)	2 months	
**Urine**			
Kovalenko et al., 1965 [29]	Urine (4 °C)	60 days	In vivo assay
Davies et al., 2017 [36]	Urine (4° C, 12 °C, 21 °C, 37 °C)	Estimated survival: 15.3/7.5/4.8/2.9 days	PCR, virus isolation
**Faeces**			
Kovalenko et al., 1965 [29]	Faeces (4 °C)	160 days	In vivo assay
Blome and Dietze 2011 [33]	Faeces (18–22 °C)	3 months	Virus isolation
de Carvalho Ferreira et al., 2014 [41]	Faeces (5 °C, 12 °C, 20 °C, 30 °C)	Half-life: 1568/660/21.7/15.3 days	PCR
Davies et al., 2017 [36]	Faeces (4 °C, 12 °C, 21 °C, 37 °C)	Estimated survival: 8.5/6.5/5.1/3.7 days	PCR, virus isolation
**Pork Products**			
McKercher et al., 1978 [42]	Pepperoni sausage	8 days	In vivo assay
McKercher et al., 1987 [6]	Muscle of Parma ham	183 days	Virus isolation (HAT)
	Bone marrow of Parma ham	94 days	
	Fat of ham	183 days	
	Pooled muscle, bone marrow, fat of ham	291 days	In vivo assay
Mebus et al., 1993 [8]	Muscle of dry cured Iberian ham/shoulder/loin/dry cured Serrano ham	112/112/98/112 days	In vivo assay
	Fat of dry cured Iberian ham/shoulder/dry cured Serrano ham	112/84/112 days	
	Bone marrow of dry cured Iberian ham/shoulder/dry cured Serrano ham	112/84/112 days	
	Lymph node of dry cured Iberian ham/dry cured Serrano ham	112/56 days	
Kolbasov et al., 2011 [5]	Meat and fat (22–27 °C, salted)	16 days	PCR
	Pork products (4–6 °C)	84 days	
	Pork products (−18–20 °C)	118 days	
Petrini et al., 2019 [7]	Italian salami	18 days	In vivo assay
	Pork belly	60 days	
	Loin	83 days	

**Table 2:** PCR and HAT results of blood, tissues and excretions on day 0. Samples highlighted in grey were also positive for infectious virus. Samples of DP1 and WB1 were stored at −20 °C, of DP2 and WB2 at 4 °C and of DP3 and WB3 at room temperature.

Sample	ASFV Genome Copies (log_10_)					
	DP1	DP2	DP3	WB1	WB2	WB3
Blood	4.73	5.98	4.40	5.50	6.98	6.50
Bone marrow	4.17	3.27	3.34	3.17	3.95	3.04
Muscle	3.10	3.31	2.90	3.92	3.79	3.36
Skin	3.93	3.47	3.37	4.51	4.49	3.95
Spleen	4.91	4.79	6.10	5.87	5.79	5.26
Faeces	1.97	1.88	neg.	3.62	3.47	2.18
Urine	3.07	2.47	neg.	3.38	3.91	2.30

## Supplementary Materials

The following are available online at https://www.mdpi.com/1999-4915/12/10/1118/s1, Figure S1: ASFV genome copy numbers as determined by qPCR in blood, spleen and bone marrow, Figure S2: ASFV genome copy numbers as determined by qPCR in muscle, skin and faeces, Figure S3: Storage of carcass material under different conditions, Table S1: Summary of raw data from qPCR (as Cq-values) and HAT of all investigated samples during storage under various conditions over time.
