# Supplementary material for: Stability of African Swine Fever Virus in Carcasses of Domestic Pigs and Wild Boar Experimentally Infected with the ASFV “Estonia 2014” Isolate
Source: Viruses. 2020 Oct 1;12(10):1118. doi: 10.3390/v12101118 (PMC7600355; doi:10.3390/v12101118)
Supplement: Supplementary file 1 [file viruses-12-01118-s001.zip › Supplement/Figures S1-3.docx]

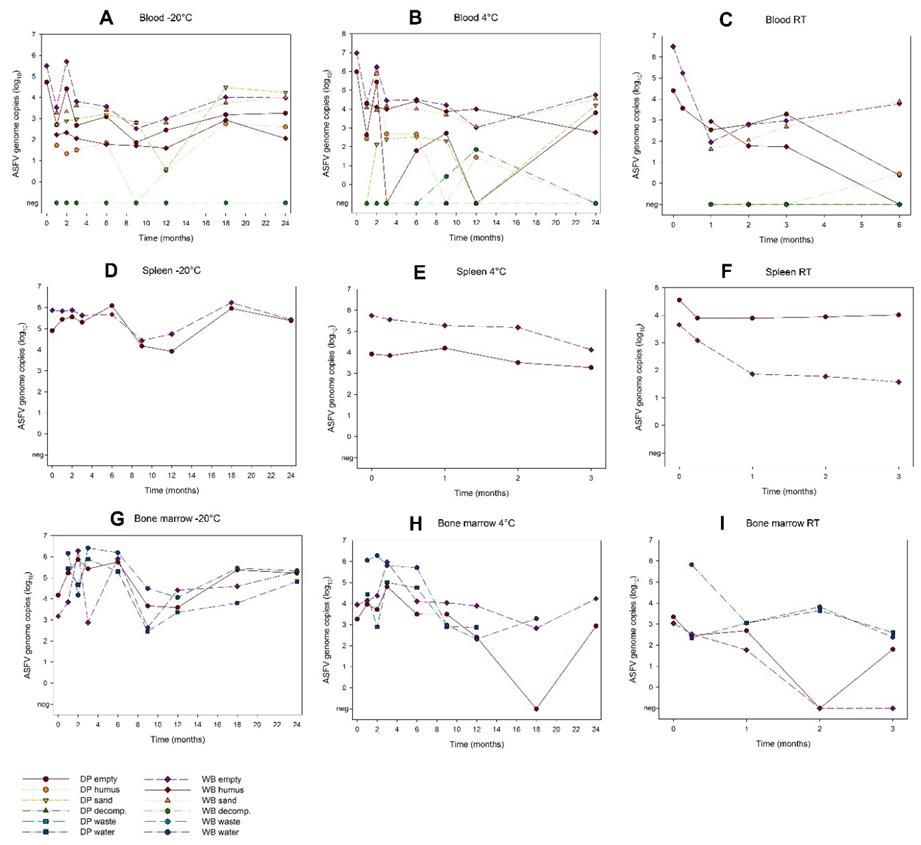


**Figure S1:** ASFV genome copy numbers as determined by qPCR in blood (A, B, C), spleen (D, E, F) and bone marrow (G, H, I) of domestic pigs (DP) and wild boar (WB) during storage in empty boxes or on different matrices (humus, sand, decomposition island [decomp.], waste, water) at -20°C, 4°C and room temperature (RT). Individual data points were connected by lines for better overview but do not represent a course.


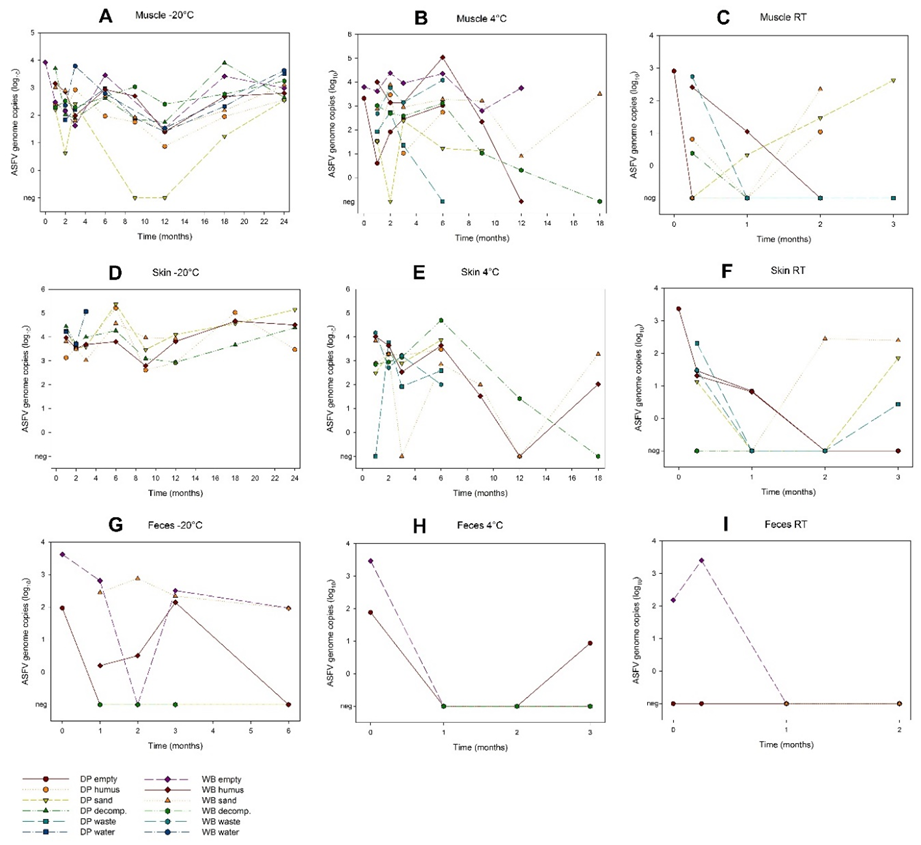


**Figure S2:** ASFV genome copy numbers as determined by qPCR in muscle (A, B, C), skin (D, E, F) and faeces (G, H, I) of domestic pigs (DP) and wild boar (WB) during storage in empty boxes or on different matrices (humus, sand, decomposition island [decomp.], waste, water) at -20°C, 4°C and room temperature (RT). Individual data points were connected by lines for better overview but do not represent a course.

**
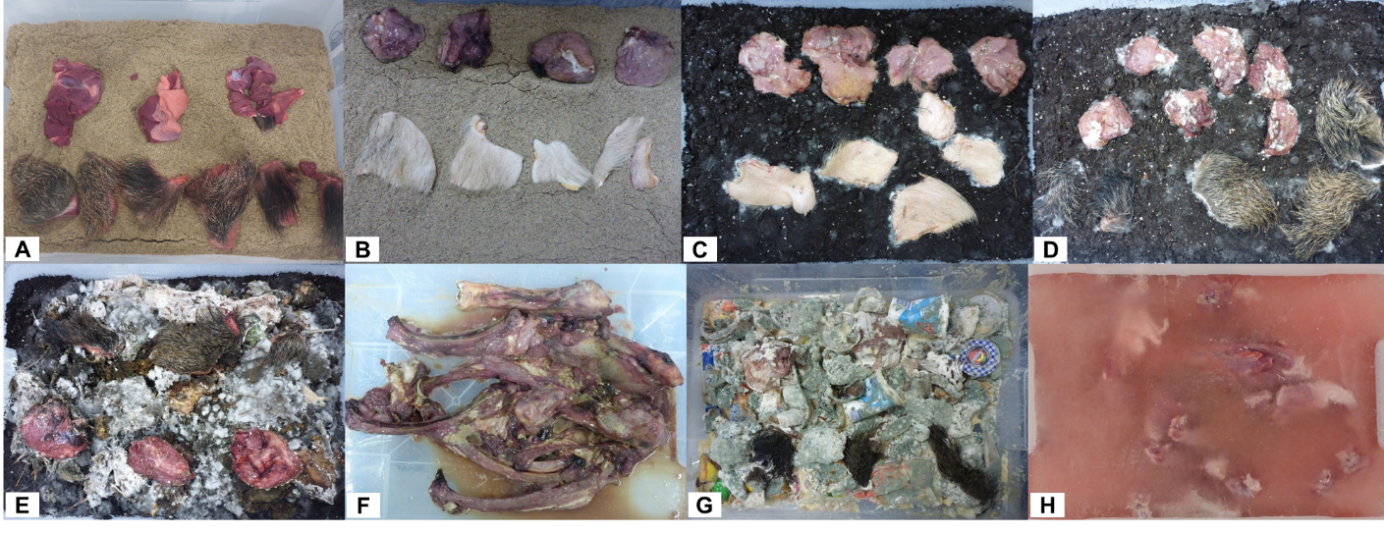
**

**Figure S3:** Storage of carcass material under different conditions. **(A)** Wild boar muscle and skin on sand on day 0. **(B)** Domestic pig muscle and skin on sand at room temperature (RT) after one week. **(C)** Domestic pig muscle and skin on humus at RT after one week. **(D)** Wild boar muscle and skin on humus at RT after one week. **(E)** Wild boar muscle and skin on soil of the decomposition island at RT after one week (note the mould growth). **(F)** Wild boar bones in an empty box at RT after one week. **(G)** Wild boar muscle and skin on waste at RT after one week (note the mould growth). **(H)** Wild boar bones in water at -20°C after one month.
