# Supplementary material for: Stability of African Swine Fever Virus in Carcasses of Domestic Pigs and Wild Boar Experimentally Infected with the ASFV “Estonia 2014” Isolate
Source: Viruses. 2020 Oct 1;12(10):1118. doi: 10.3390/v12101118 (PMC7600355; doi:10.3390/v12101118)
Supplement: Supplementary file 1 [file viruses-12-01118-s001.zip › Supplement/Table S1.docx]

**Table S1:** Summary of raw data from qPCR (as Cq-values) and HAT of all investigated samples during storage under various conditions over time. **Animals**: All samples except spleens were stored as follows: DP1 and WB1 at -20°C; DP2 and WB2 at 4°C; DP3 and WB3 at room temperature. All six spleens were stored at -20°C during the entire study period. **Sampled tissues and fluids**: Blood, bone marrow (bone), brain, faeces, muscle (musc), skin, spleen, urine. **Sampled matrices**: Humus, sand, soil of the decomposition island (sampled underneath the pieces of muscle or skin), water (in which bones, muscle or skin were stored). **Matrices** on which the body tissues were stored: Humus, sand, soil of the decomposition island (decomp), waste, water. HAT results of bone marrow marked with * are red bone marrow, otherwise yellow bone marrow was tested. For HAT evaluation positive (pos) and negative (neg) results were differentiated.
